# Oral Exposure to Microplastics Affects the Neurochemical Plasticity of Reactive Neurons in the Porcine Jejunum

**DOI:** 10.3390/nu16142268

**Published:** 2024-07-14

**Authors:** Ismena Gałęcka, Jarosław Całka

**Affiliations:** 1 Department of Epizootiology, Faculty of Veterinary Medicine, University of Warmia and Mazury in Olsztyn, Oczapowskiego 13, 10-719 Olsztyn, Poland; 2 Department of Clinical Physiology, Faculty of Veterinary Medicine, University of Warmia and Mazury in Olsztyn, Oczapowskiego 13, 10-719 Olsztyn, Poland

**Keywords:** enteric nervous system, microparticles, plastic, polyethylene terephthalate, small intestine, gastrointestinal tract, food chain

## Abstract

Plastics are present in almost every aspect of our lives. Polyethylene terephthalate (PET) is commonly used in the food industry. Microparticles can contaminate food and drinks, posing a threat to consumers. The presented study aims to determine the effect of microparticles of PET on the population of neurons positive for selected neurotransmitters in the enteric nervous system of the jejunum and histological structure. An amount of 15 pigs were divided into three groups (control, receiving 0.1 g, and 1 g/day/animal orally). After 28 days, fragments of the jejunum were collected for immunofluorescence and histological examination. The obtained results show that histological changes (injury of the apical parts of the villi, accumulations of cellular debris and mucus, eosinophil infiltration, and hyperaemia) were more pronounced in pigs receiving a higher dose of microparticles. The effect on neuronal nitric oxide synthase-, and substance P-positive neurons, depends on the examined plexus and the dose of microparticles. An increase in the percentage of galanin-positive neurons and a decrease in cocaine and amphetamine-regulated transcript-, vesicular acetylcholine transporter-, and vasoactive intestinal peptide-positive neurons do not show such relationships. The present study shows that microparticles can potentially have neurotoxic and pro-inflammatory effects, but there is a need for further research to determine the mechanism of this process and possible further effects.

## 1. Introduction

The jejunum is the section of the small intestine that has the highest length in relation to other sections of the digestive tract. This is where the essential digestion and absorption of nutrients takes place. Its proper function is mediated inter alia by the enteric nervous system (ENS), which regulates food intake, enzyme and electrolyte secretion, blood flow, and immune response [1,2,3]. It is made up of a dense network of nerve connections that are concentrated in two main plexuses. The myenteric plexus (Auerbach’s plexus) is located between the circular and the longitudinal layers of smooth muscle along the entire length of the gastrointestinal tract, i.e., from the oesophagus to the anus [4,5]. The submucous plexus (Meissner’s plexus) is located in the mucous membrane, mainly in the small and large intestine [1,3,4,5,6]. The number of plexuses is determined by the animal species and the section of the digestive tract [3,5].

Both plexuses exhibit the capacity to regulate the secretory and motor functions of the gastrointestinal tract [3,7]. The myenteric plexus (MP) is mainly responsible for the passage of food content through the digestive tract [7]. The inhibitory neurotransmitters, responsible for the relaxation of the intestinal muscle layer, mainly include vasoactive intestinal peptide (VIP), nitric oxide (NO), pituitary adenylate cyclase-activating peptide (PACAP), and purines, whereas the stimulatory action is mainly exhibited by tachykinins (including, e.g., neurokinin A (NKA), substance P (SP)), acetylcholine (ACh), or galanin (GAL) [4,8]. In turn, the submucous plexus neurons mainly regulate epithelial secretory functions and blood flow [2,7]. In pigs, the situation is similar to humans; the submucous plexus can be divided into two parts, namely the outer submucous plexus (OSP) located near the circular muscle, and the inner submucous plexus (ISP) located near the lamina propria of the mucous membrane [3,6]. VIP and ACh are the main neurotransmitters of the secretomotor neurons of this plexus [9]. Similar to MP, ACh mainly exhibits excitatory action, whereas NO exhibits inhibitory action on the smooth muscle as well as the epithelial and enteroendocrine cells [6].

The digestive tract is one of the barriers against the external environment. In addition to food, bacteria, viruses, and contaminants such as heavy metals or plastic microparticles can enter the body. The latter have been recently posing a potential threat to humans, animals, or the natural environment [10,11,12,13]. Available data indicate that only approximately 9% of plastics are recycled, with the remainder penetrating into the natural environment [12], where, under the influence of chemical and physical factors, nano- and microparticles are formed. Pinlova et al. estimate that up to 480 million particles can be formed from a 0.5 litre PET (polyethylene terephthalate) bottle [14], which is likely to be a significant risk factor later on. This is continuing to emerge, as microplastics are ubiquitous [12,15,16,17,18]. Determining the exact daily dose to which a person standard consumer is exposed is difficult to evaluate. It depends on several factors, such as dietary preferences and age. It should also be taken into account that not only the alimentary route, but also, for example, the inhalation of microplastics from the air can represent a gateway of entry into the living organism. Senathirajah et al. estimated the exposure to microplastics to be between 0.1 and 5 g of microplastics/week/person [19], which translates into an average of between 0.2 and 10.2 mg/kg of body weight/day [20]. To date, the presence of microplastics in humans has been confirmed in faeces, colon, placenta, breast milk, lungs, bronchoalveolar fluid, sputum, blood, spleen, liver, and kidneys [13,17,21]. The oral and inhalation routes, and, to a lesser extent, the dermal route, are considered to be the main routes of exposure in humans [13,18,21].

As plastics are widely used in the agri-food sector, the food chain is a critical point of consumers’ exposure to its nano- and microparticles. Feed may be a source of pigs’ exposure to microplastics [22], as evidenced by their subsequent occurrence in their faeces [23]. The processing of food products [24], or storing them in containers made of plastic, can contribute to the entry of microparticles into the body [12,18,25]. The microplastics chosen for the experiment were polyethylene terephthalate (MP-PET) that are used in the food industry for packing food and beverages. In addition, the studies presented in the available literature are dominated by other types of plastics [17,26,27,28,29], which is why the investigation of the impact of PET fills an existing knowledge gap.

The aim of the current study was to determine the percentage of neurons positive in relation to selected neuronally active substances (cocaine- and amphetamine-regulated transcript (CART), galanin (GAL), neuronal nitric oxide synthase (nNOS), substance P (SP), vesicular acetylcholine transporter (VAChT), and vasoactive intestinal peptide (VIP)) and histological changes in the jejunum of the domestic pig after a 28-day exposure to MP-PET at a dose of 0.1 g/day/animal and 1 g/day/animal in order to better understand the effects of microplastics on the ENS. Previous results regarding a different part of the gastrointestinal tract study from the same animals were published by Gałęcka et al. [30].

## 2. Materials and Methods

### 2.1. Animals

**Figure 1:**
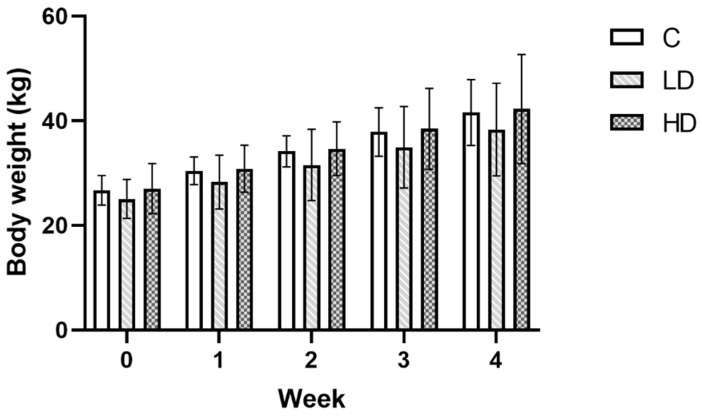
Bodyweight of gilts in weekly measurements. There were no statistically significant differences between groups in weeks 0, 1, 2, 3, and 4 (*p* > 0.05).

The study used 15 sexually immature Pietrain × Duroc gilts from a farm in Lubawa (Poland), which weighed approximately 20 kg (Figure 1) and were eight weeks old at the start of the experiment. The animals were kept in the premises of the Faculty of Veterinary Medicine of the University of Warmia and Mazury in Olsztyn under the following conditions: a temperature of 20–22 °C, relative humidity of 55–60%, and a 12 h light–dark cycle. The animals were fed twice a day with commercial feed and had access to water ad libitum. Before introducing the animals, plastic objects were removed from the surroundings. Feeders were made from stainless steel. Before the experiment started, a 7-day acclimatisation in the new environment was carried out. The gilts were randomly divided into three study groups of five individuals already at the acclimatisation stage. Animals were randomised using a computer based random order generator. The handling of the animals and all the procedures were carried out in accordance with Polish laws (Journal of Laws 2015, No 266), which lay down the conditions and methods for carrying out experiments on animals, and Directive 2010/63/EU of the European Parliament and of the Council of 22 September 2010 on the protection of animals used for scientific purposes. The experiment protocols were submitted to and approved by the Local Scientific Research Ethics Committee of the University of Warmia and Mazury in Olsztyn (Decision No. 10/2020 of 26 February 2020).

### 2.2. Experimental Procedures

**Figure 2:**
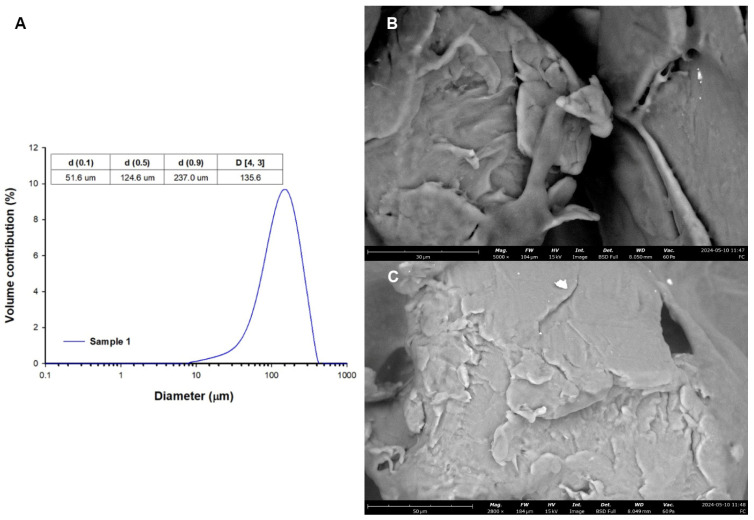
(**A**) Particle size distribution and (**B**,**C**) SEM images.

The gilts were divided into three groups: a control group (C; *n* = 5), a group which received a low dose (LD; *n* = 5) of 0.1 g of MP-PET/day/animal, and a group which received a high dose (HD; *n* = 5) of 1 g of MP-PET/day/animal for 28 days. Animal handling always started with group C, then LD, and then HD. The animal keeper was not aware of the treatment group allocation. The microparticles were administered once daily, orally by capsule applicator, one hour before the morning feeding, in gelatine capsules. Animals in group C were administered empty capsules, whereas those in groups LD and HD were administered capsules with microparticles of polyethylene terephthalate (Cat. No ES306031/1, Goodfellow Cambridge Ltd., Huntingdon, UK). The MP-PET particles were heterogeneously shaped. Particle size was determined using laser diffraction analysis with a Mastersizer 2000 analyzer (Malvern Instruments Ltd., Malvern, UK). The obtained results indicate that the analysed sample contains particles in the range of 7.6 µm–416.9 µm, with the dominant share of particles with a diameter of 158.5 µm. Analysis of the results shows that in this sample 10% of particles have diameters no greater than 51.6 µm, 50% have diameters no greater than 124.6 µm, and 90% have diameters smaller than 237.0 µm. The mean diameter of particles D [3,4] in the sample is 135.6 µm Figure 2A. Microscopic analysis of particles was performed using a Phenom ProX G6 scanning microscope (ThermoFisher Scientific, Waltham, MA, USA) (Figure 2B,C). The applied dose of 0.1 g/day/animal was adjusted, based on a study by Deng et al. [31], to the weight of the gilts, whereas the dose of 1 g/day/animal was a 10-fold higher dose (calculated on a logarithmic scale—the logarithm of the decimal (for log(0.1)  =  −1; for log(1)  =  0)), and was of an overloading nature.

### 2.3. Tissue Sampling

After 28 days, the animals were euthanised. The euthanasia protocol was based on the use of atropine (0.05 mg/kg i.m., Polfa, Warszawa, Poland), followed by xylazine (3 mg/kg i.m., Vet-Agro, Lublin, Poland) and ketamine (6 mg/kg i.m., Vetoquinol Biowet, Gorzów Wielkopolski, Poland). After approximately 20 min, when the gilts were unconscious, an overdose of sodium pentobarbital (0.6 mL/kg i.v., Biowet, Puławy, Poland) was applied. Once the cessation of vital functions was confirmed (no pulse, no respiration, no corneal reflex, cardiac rhythm disappeared, and respiration ceased), sampling for further tests began immediately. An approximately 8-cm-long section of the jejunum was taken, a section located 100 cm from the pylorus of the stomach. The tissues were immediately fixed in order to carry out double immunofluorescence staining and histological examination.

### 2.4. Double Immunofluorescence Staining

In order to fix the samples, the collected sections of the jejunum were placed in a 4% paraformaldehyde solution (pH 7.4) for one hour and then rinsed for three consecutive days with 0.1 M phosphate buffer (pH 7.4), with the buffer being changed every 24 h. After three days, the tissues were transferred to an 18% sucrose solution (pH 7.4) for two weeks at a temperature of 4 °C. Once fixed, sections of the jejunum were embedded in OCT Tissue-Tek (Sakura Finerek USA, Inc., Torrance, CA, USA) and, using a cryostat (CM 1860, Leica, Nussloch, Germany), cut at a temperature of −22 °C into sections with a thickness of 14 µm, and attached to chrome-alum-coated slides. The staining procedure lasted for two days, (1) the slides being first dried at room temperature for 45 min, (2) followed by rinsing with 0.1 M phosphate-buffered saline (PBS, pH 7.4, 3 × 15 min). (3) The next step involved incubation with a blocking mixture (10% horse serum and 0.1% bovine serum albumin in 0.1 M PBS, 1% Triton X-100, 0.05% Thimerosal, and 0.01% sodium azide) for 1 h at room temperature in a humid chamber. (4) The blocking mixture was washed out using PBS solution (3 × 15 min). (5) A mixture of primary antibodies consisting of protein gene product 9.5 (PGP 9.5, a pan-neuronal marker, used to visualise neurons in the jejunum) and against one of the test substances (CART/GAL/nNOS/SP/VAChT/VIP) was applied onto the intestinal sections overnight. (6) On the second day of staining, the sections were washed out with PBS solution (3 × 15 min). (7) Secondary antibodies Alexa Fluor 488 (to visualise PGP 9.5 positive neurons) and 546 (to visualise CART/GAL/nNOS/SP/VAChT/VIP positive neurons) were applied onto the tissues for 1 h in a humid chamber. The list of antibodies used in immunofluorescence method is presented in Table 1. (8) The final staining step involved rinsing with PBS solution (3 × 15 min), (9) coating the tissues with carbonate-buffered glycerol (pH 8.6), and (10) covering them with a cover slip. Routine follow-up antibody tests were performed to exclude non-specific staining. These tests involved the pre-absorption of antibodies with the relevant antigen, the omission test, and the replacement of primary antibodies with non-immune sera. The stained intestinal fragments were analysed using a Zeiss Axio Imager.M2 fluorescence microscope (Zeiss, Oberkochen, Germany) equipped with the appropriate set of filters. At least 500 PGP 9.5-positive neurons (green) with a clearly visible nucleus in each of the three plexuses (MP, OSP, and ISP) were counted as 100% of the population. By changing the filter, the neurons positive to the test substances (red) were then visualised, counted, and determined as a percentage of PGP 9.5-positive neurons. In order to avoid counting the same neurons, the distance between the fragments was at least 200 µm.

### 2.5. Histological Examinations

The samples of jejunum were fixed in 4% paraformaldehyde solution in 0.1 M phosphate buffer (pH 7.4) for 48 h, dehydrated in ethanol in a tissue infiltration machine (Leica TP1020, Leica, Nussloch, Germany), and embedded in paraffin (EG1150, Leica, Nussloch, Germany). The 4 µm-thick sections were prepared with microtome (HM 340E, Microm, Spain) and stained with the haematoxylin and eosin method (HE) using a multistainer (ST5020 + CV5030, Leica, Nussloch Germany). The slides were digitalised in a Pannoramic 250 Flash scanner (3DHistech, Budapest, Hungary). Morphometric analyses were performed using SlideViewer 2.6 software (3DHistech, Budapest, Hungary) and were included the length of villi, the crypt depth, the thickness of mucosa, the thickness of submucosa, and the thickness of muscularis externa. The measurements were performed on three sections per animal, separated each other by at least 1 cm, in 10 replicates per slide, and the mean values were subjected to a statistical analysis.

### 2.6. Statistical Analysis

Prior to statistical analysis, the assumption of linearity and normality was checked. For linearity testing, two-dimensional scatter plots of the analysed variables were generated. The assumption of normality was confirmed using histograms and residual normality plots. The following values were calculated from the results obtained as follows: the mean (M), the standard deviation (SD), and the standard error of the mean (SEM). One-way analysis of variance (ANOVA) was applied to demonstrate statistically significant differences between the number of neurons immunoreactive to CART, GAL, nNOS, SP, VAChT, and VIP (dependent variable) in each study group (qualitative variable).

Analysis of variance was used for statistically significant indices between the morphometric indices (dependent variables) in individual study groups (qualitative variable). The homogeneity of variance was determined using Levene’s test before the ANOVA. A post hoc analysis (Scheffe’s test) was used to assess statistically significant differences between individual study groups. The differences were considered statistically significant at *p* < 0.05 (* *p* < 0.05, ** *p* < 0.01, *** *p* < 0.001). A statistical analysis of the results was conducted using Statistica 13.3 software (TIBCO Software Inc., Palo Alto, USA), whereas the visualisation was carried out using GraphPad Prism 9.0.0 software (GraphPad Software, Boston, MA, USA).

## 3. Results

### 3.1. Myenteric Plexus

**Figure 3:**
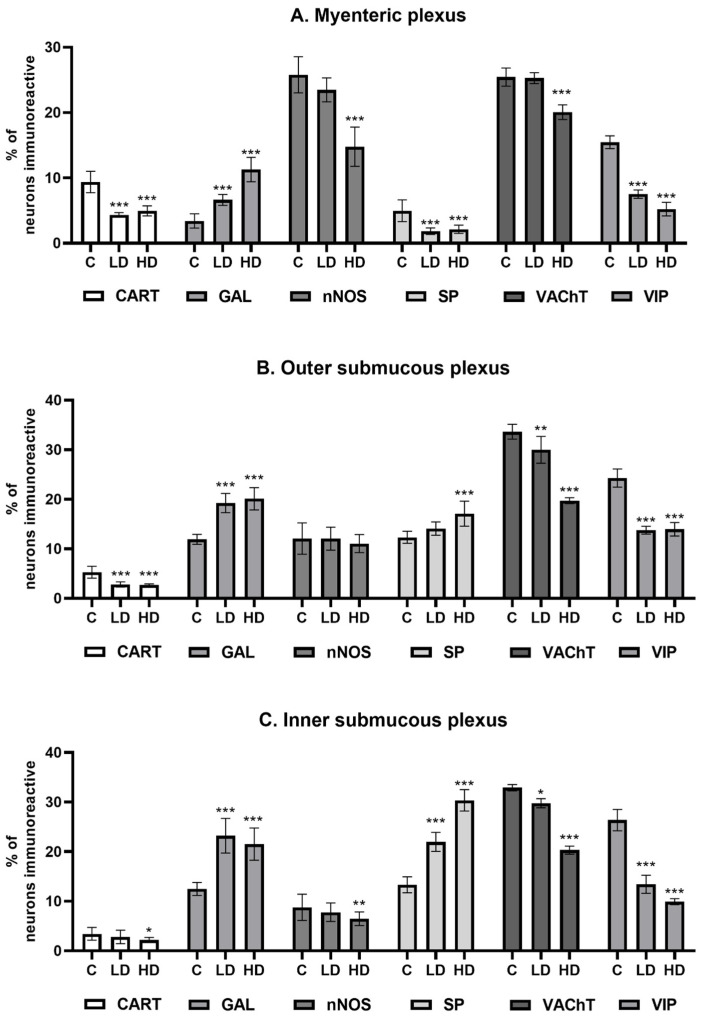
Percentage of neurons immunoreactive (**A**) in the myenteric plexus, (**B**) outer submucous plexus, and (**C**) inner submucous plexus. Percentage (Mean ± SEM) of neurons immunoreactive for cocaine- and amphetamine-regulated transcript peptide (CART), galanin (GAL), neuronal nitric oxide synthase (nNOS), substance P (SP), vesicular acetylcholine transporter (VAChT), and vasoactive intestinal polypeptide (VIP) of all neuronal cells immunoreactive to PGP 9.5 in the control group (**C**), low-dose group (LD), and high-dose group (HD) of MP-PET. * *p* < 0.05, ** *p* < 0.01, *** *p* < 0.001 indicate statistically significant differences in the expression of the tested substances in relation to the control group.

**Figure 4:**
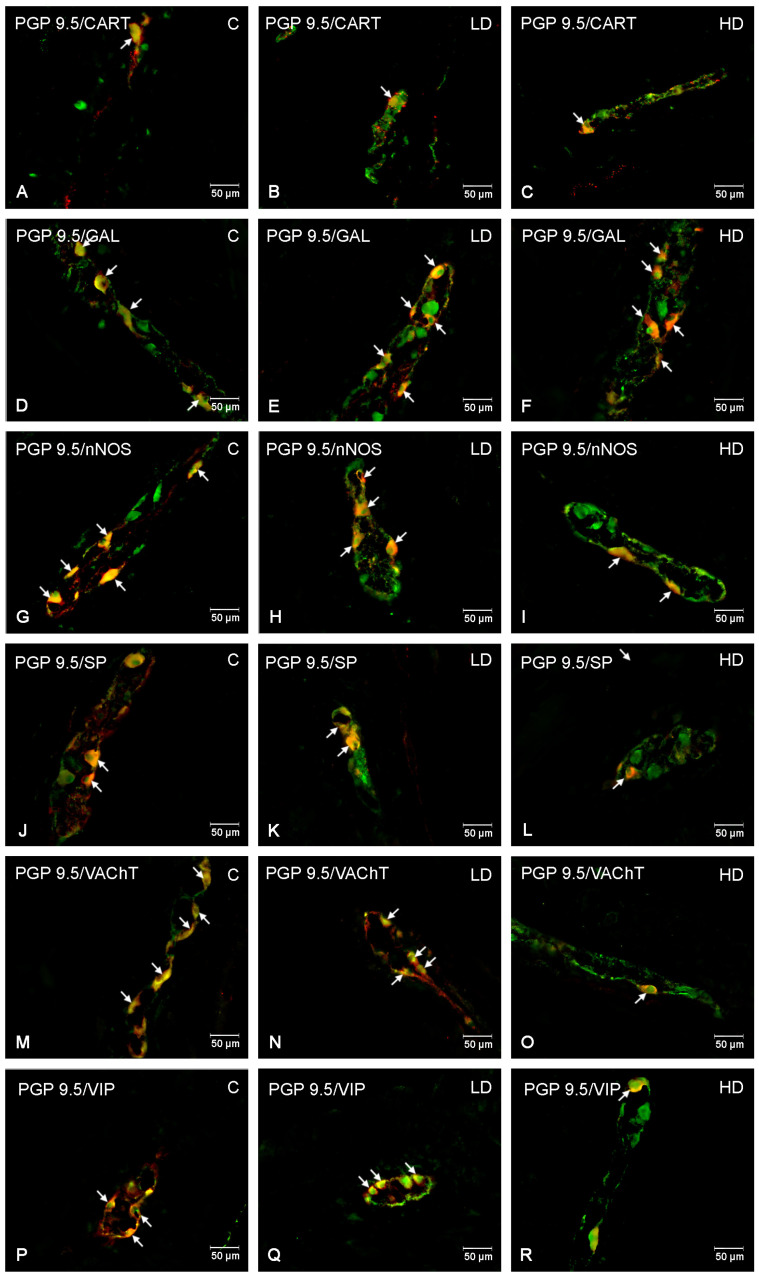
The myenteric plexus of porcine jejunum. Fluorescence microscope image showing the distribution of neurons immunoreactive to PGP 9.5—used as a pan-neuronal marker and CART, GAL, nNOS, SP, VAChT, VIP in the control group—C (**A**,**D**,**G**,**J**,**M**,**P**), after administration of low (**B**,**E**,**H**,**K**,**N**,**Q**) and high doses (**C**,**F**,**I**,**L**,**O**,**R**) of MP-PET. All photographs were made by overlapping green and red fluorescent channels (green for PGP 9.5 and red for CART, GAL, nNOS, SP, VAChT, and VIP, respectively). Neurons immuno-positive to a particular substance studied are indicated with arrows.

Under physiological conditions, the most abundant population of all the tested substances was that of nNOS-positive neurons, standing at 25.75 ± 2.78% (C), which decreased to 14.77 ± 3.02% under the influence of HD MP-PET. Cholinergic neurons represented by VAChT 25.45 ± 1.39% (C), the percentage of which also decreased to 20.06 ± 1.13% under the influence of HD, were comparably abundant. LD MP-PET did not contribute to the emergence of statistically significant differences. LD and HD MP-PET also reduced the population of CART-positive neurons from 9.38 ± 1.64% (C) to 4.32 ± 0.37% (LD) and 4.95 ± 0.77% (HD), the population of VIP-positive neurons from 15.46 ± 0.98% (C) to 7.50 ± 0.64% (LD) and 5.23 ± 1.04% (HD), and that of SP-positive neurons from 4.97 ± 1.66% (C) to 1.83 ± 0.50% (LD) and 2.13 ± 0.62% (HD). An increase in the neuronal population only occurred for GAL, where an increase from 3.40 ± 1.08% (C) to 6.63 ± 0.83% (LD) and 11.28 ± 1.86% (HD) could be observed. It should be noted that only the MP showed a reduction in the percentage of SP-positive neurons, in contrast to the two submucous plexuses, where an increase occurred. The data are provided in Figure 3A and Figure 4.

### 3.2. Outer Submucous Plexus

**Figure 5:**
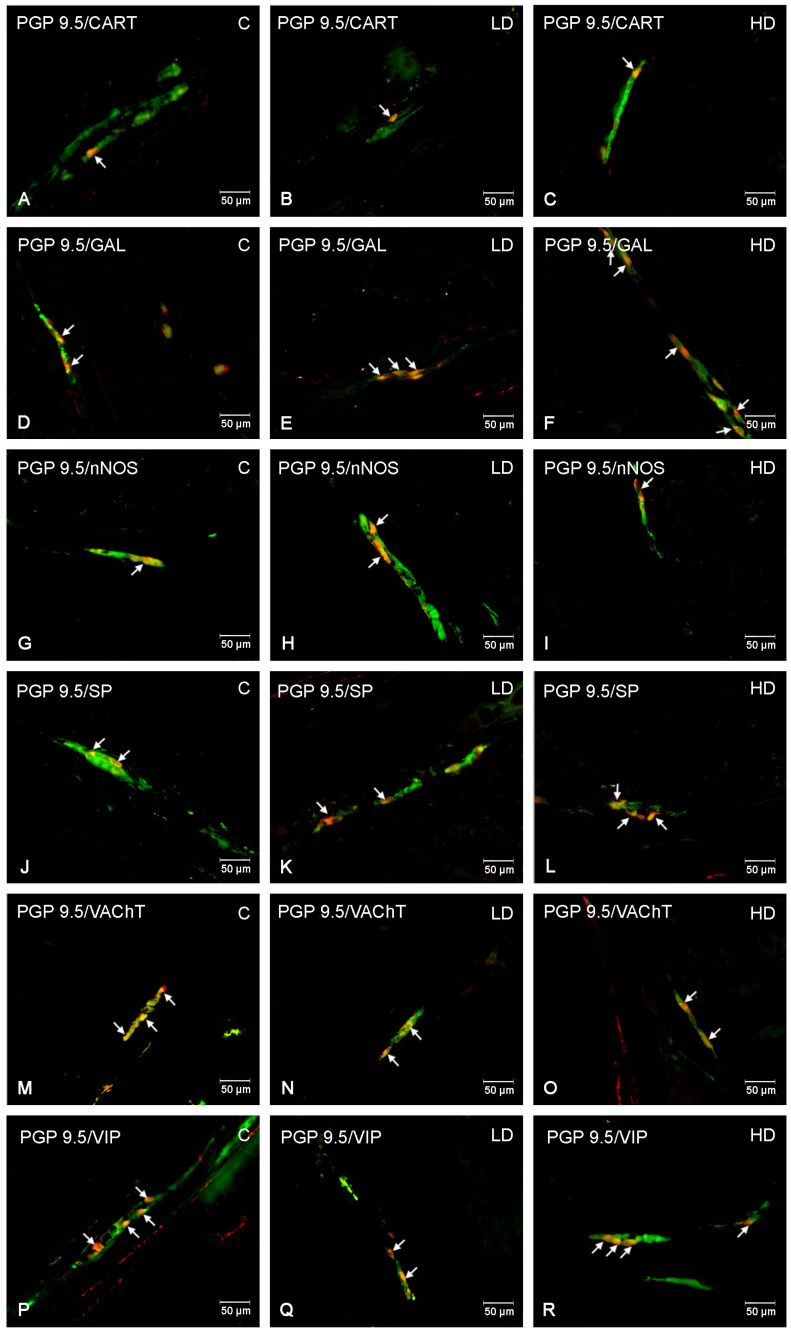
The outer submucous plexus of porcine jejunum. Fluorescence microscope image showing the distribution of neurons immunoreactive to PGP 9.5 —used as a pan-neuronal marker and CART, GAL, nNOS, SP, VAChT, and VIP in the control group—C (**A**,**D**,**G**,**J**,**M**,**P**), after administration of low (**B**,**E**,**H**,**K**,**N,Q**) and high doses (**C**,**F**,**I**,**L**,**O**,**R**) of MP-PET. All photographs were made by overlapping green and red fluorescent channels (green for PGP 9.5 and red for CART, GAL, nNOS, SP, VAChT, and VIP, respectively). Neurons immuno-positive to a particular substance studied are indicated with arrows.

Cholinergic neurons and VIP-positive neurons jointly accounted for almost 58% of all neurons in the OSP under physiological conditions. In both cases, following exposure to MP-PET, a decrease was observed in the percentage of VAChT-positive neurons from 33.63 ± 1.53% (C) to 29.99 ± 2.69% (LD) and 19.71 ± 0.60% (HD), and for VIP-positive neurons from 24.28 ± 1.84% (C) to 13.77 ± 0.78% (LD) and 13.95 ± 1.38% (HD). The population of CART-positive neurons also decreased from 5.27 ± 1.20% (C) to 2.80 ± 0.53% (LD) and 2.67 ± 0.26% (HD). The percentage of GAL-positive neurons increased from 11.90 ± 1.00% (C) to 19.24 ± 1.93% (LD) and 20.10 ± 2.25% (HD). The population of SP-positive neurons increased from 12.32 ± 1.22% (C) to 17.10 ± 2.52% (HD). No statistically significant differences induced by LD MP-PET were observed. Interestingly, neither LD nor HD MP-PET contributed to a change in the population size of nNOS-positive neurons. The data are provided in Figure 3B and Figure 5.

### 3.3. Inner Submucous Plexus

**Figure 6:**
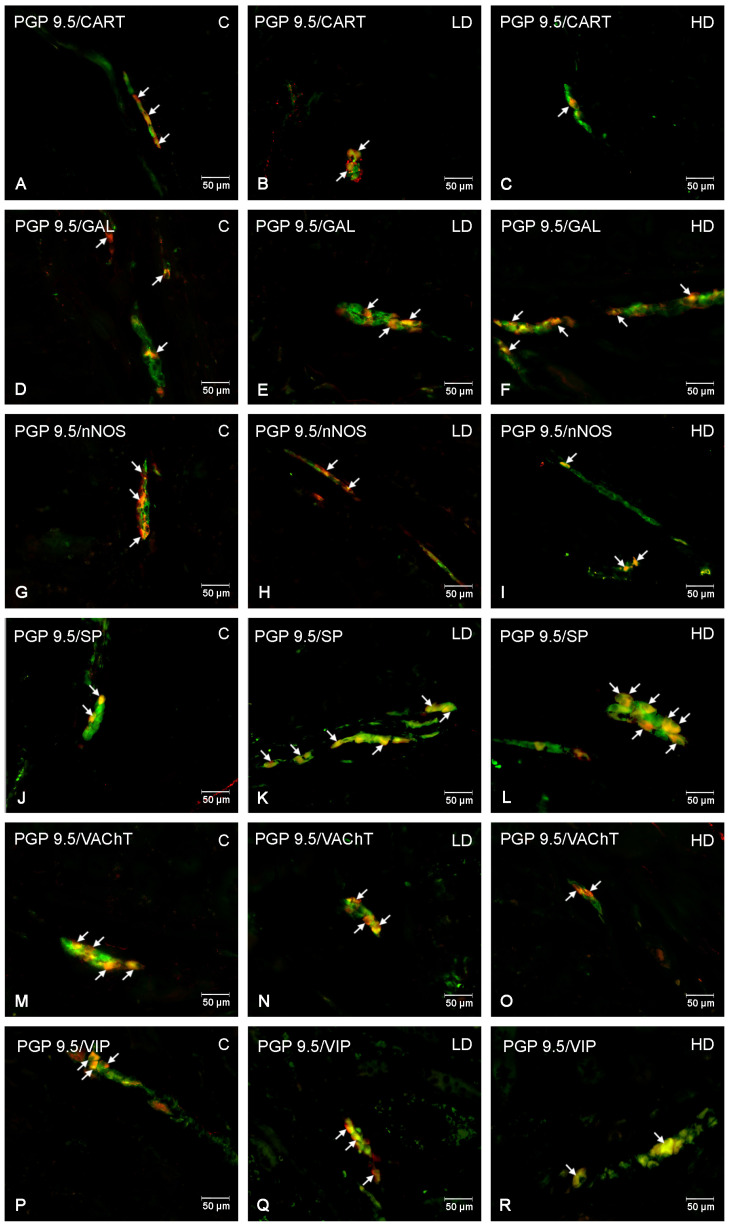
The inner submucous plexus of porcine jejunum. Fluorescence microscope image showing the distribution of neurons immunoreactive to PGP 9.5 —used as a pan-neuronal marker and CART, GAL, nNOS, SP, VAChT, and VIP in the control group—C (**A,D,G,J,M,P**), after administration of low (**B**,**E**,**H**,**K**,**N**,**Q**) and high doses (**C**,**F**,**I**,**L**,**O**,**R**) of MP-PET. All photographs were made by overlapping green and red fluorescent channels (green for PGP 9.5 and red for CART, GAL, nNOS, SP, VAChT, and VIP, respectively). Neurons immuno-positive to a particular substance studied are indicated with arrows.

Similar to the previously described plexus, VIP- and VAChT-positive neurons represented the most abundant population under physiological conditions. The population of VAChT-positive neurons decreased from 32.91 ± 0.63% (C) to 29.76 ± 0.91% (LD) and 20.30 ± 0.82% (HD), whereas that of VIP-positive neurons decreased from 26.37 ± 2.14% (C) to 13.41 ± 1.84% (LD) and 9.92 ± 0.60% (HD). A more than two-fold increase in SP-positive neurons under the influence of HD, from 13.30 ± 1.60% (C) to 30.35 ± 2.15% (HD), was noted. The low dose contributed to an increase in the population of these neurons, but to a lesser extent, being 21.96 ± 1.92% (LD). An increase in the percentage of GAL-positive neurons was also noted from 12.49 ± 1.30% (C) to 23.22 ± 3.49% (LD) and 21.52 ± 3.25% (HD). LD MP-PET did not contribute to changes in the population of CART-positive and nNOS-positive neurons, in contrast to HD. The percentage of CART-positive neurons decreased from 3.42 ± 1.28% (C) to 2.19 ± 0.51% (HD), whereas the population of nitrergic neurons (nNOS) decreased from 8.76 ± 2.66% (C) to 6.48 ± 1.38% (HD). The data are provided in Figure 3C and Figure 6.

### 3.4. Histological Examinations

**Figure 7:**
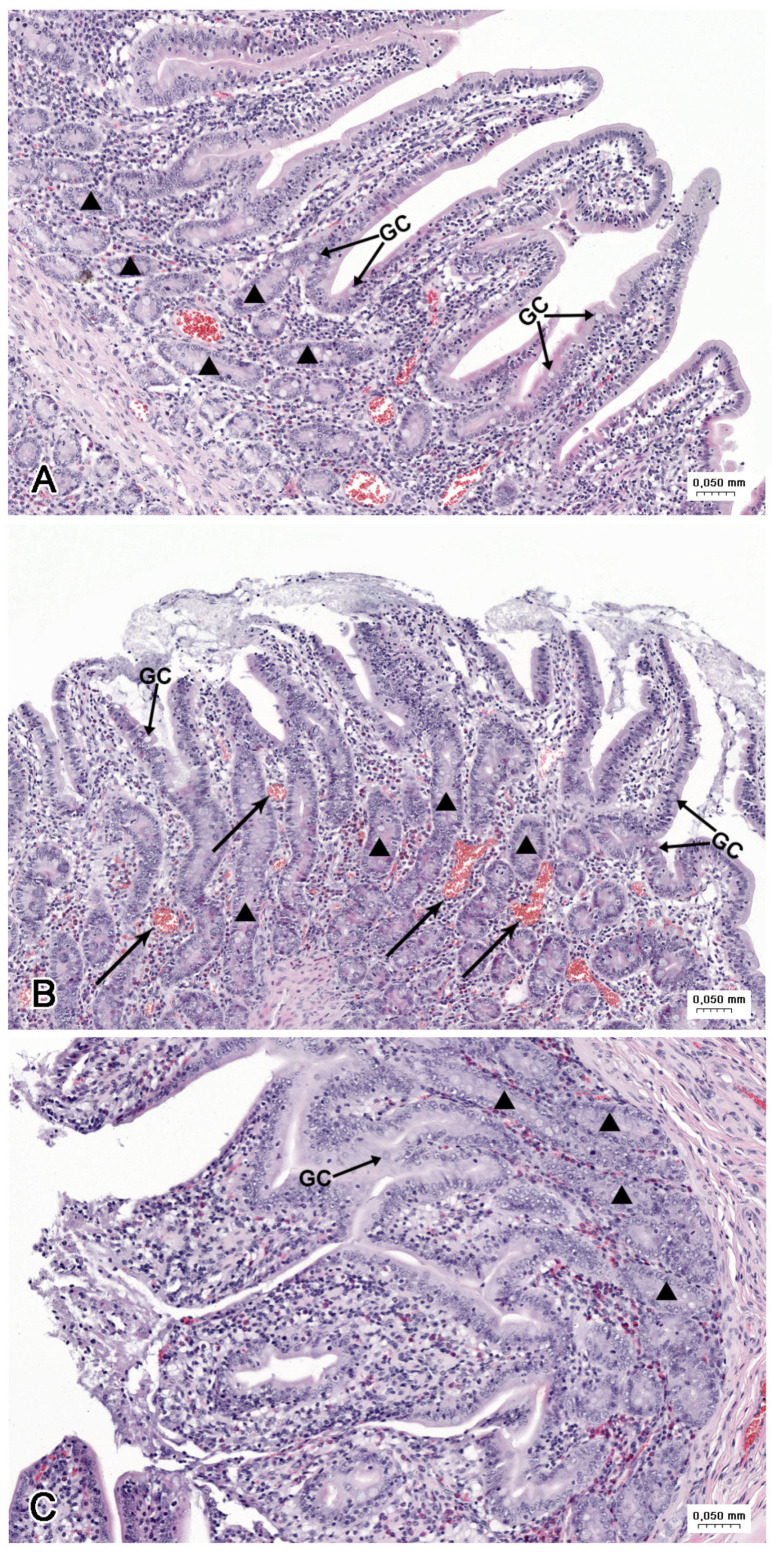
Histological structure of the jejunum (**A**) in the C group, (**B**) in the LD group, and (**C**) the HD group. (**A**) Tunica mucosa with the villi covered by continuous columnar epithelium. (**B**) Desquamation of enterocytes leading to injury of the apical parts of villi. Note the numerous blood-containing vessels (arrows). (**C**) Loss of enterocytes on the apical and lateral surfaces of the villi. GC—goblet cells, triangle—intestinal glands.

The jejunum of control animals was characterised by the presence of irregularly shaped villi of moderate length, which were covered by the columnar epithelium comprising mostly adsorptive enterocytes and much fewer numerous goblet cells (Figure 7A). The intestinal glands had the form of abundant tortuous tubes (Figure 7A). The lamina propria contained lymphocytes, plasma cells, and eosinophils. The muscularis mucosae was well developed. Villi with damaged apical parts were infrequently observed in control animals.

**Figure 8:**
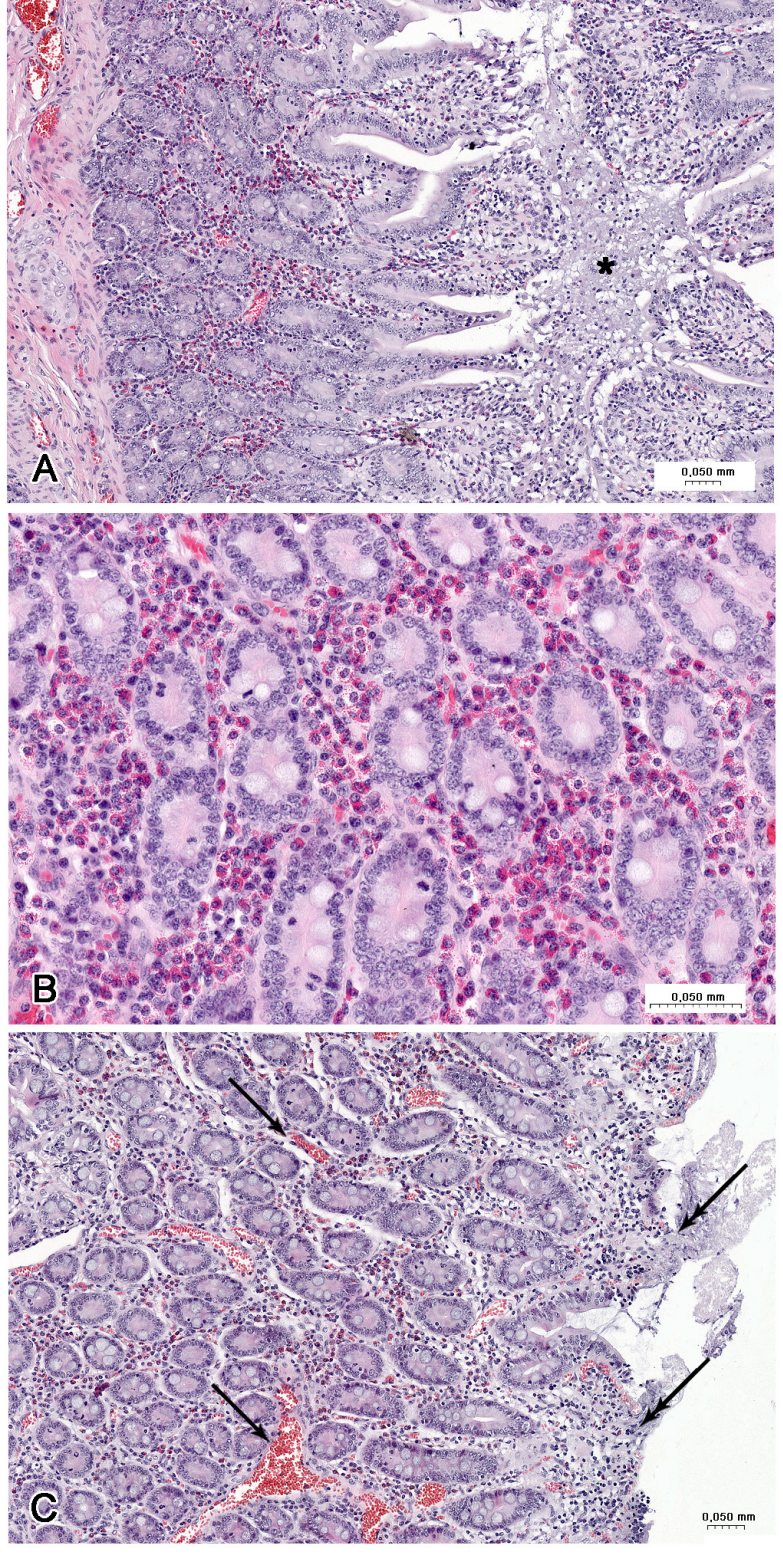
Histological structure of the jejunum (**B**) in a LD group and (**A**,**C**) HD group. (**A**) Accumulation of cellular debris and mucous (asterisk) in fold of mucosa. Note the hyperaemia and strong infiltration by eosinophils. (**B**) Numerous eosinophils in the lamina propria between the intestinal glands. (**C**) Numerous blood-containing vessels (arrows) in the lamina propria and damage of the villi (double arrows).

The desquamation of enterocytes leading to injury of the apical parts of the villi was frequently observed in the jejunum samples from the animals treated with LD (Figure 7B) and HD of MP-PET (Figure 7C). The loss of enterocytes or the presence of an abnormal epithelium was also noted on the lateral surfaces of the villi (Figure 7C). The large accumulations of cellular debris and mucus occurred in folds of the mucosa (Figure 8A). Strong eosinophil infiltration (Figure 8B) and hyperaemia (Figure 8C) were observed in the lamina propria in both experimental groups. The pathological changes were more prominent in the pigs receiving higher doses of PET.

**Figure 9:**
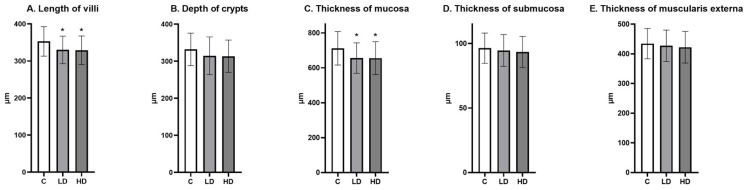
(**A**) The length of villi, (**B**) the depth of crypts, (**C**) the thickness of mucosa, (**D**) the thickness of submucosa, and (**E**) the thickness of the muscularis externa in the jejunum (Mean ± SD). * *p* < 0.05 indicate statistically significant differences in the expression of the tested substances in relation to the control group in the LD group and HD group.

The morphometric analysis showed that the length of villi and the thickness of mucosa were significantly lower in the LD and HD groups compared to the C groups. The depth of crypts, as well as the thickness of the submucosa and the muscularis externa, did not differ between the groups (Figure 9).

## 4. Discussion

The study presented in this article confirms that microplastic, as a xenobiotic, affects neuronal populations within the ENS of the jejunum. However, the mechanism of affecting neurons and other cells has not been fully understood. Neurotoxicity can be linked to the ability of particles to accumulate in the mitochondria, leading to mitochondrial dysfunction [32], the induction of oxidative stress [26,33], direct physical damage [28,34], altered neurotransmitter levels [26,30], or disruption of the gut microbiome [33,34,35], inducing pro-apoptotic protein expression (BAX, caspase 3, caspase 8, caspase 9, DR5, and cytochrome c) and pro-inflammatory cytokines (IL-8, NF-κB and TNF-α) [36]. Up to date, most studies confirm that it is the size of particles that is of greatest importance [26,37]. A size of 10 µm is considered to be the upper limit for microplastic uptake by the cells through phagocytosis, pinocytosis or macropinocytosis [38,39]. Particles larger than 150 µm can be bound to the intestinal mucus layer, whereas smaller particles are capable of penetrating this layer [40], leading to changes in the histological structure [10,34,40,41]. Since the potential effect caused by oral exposure to microplastics is determined by the plastic used, the results obtained may vary, and further research needs to be carried out using other types of microparticles as well as mixtures of different plastics. Another factor influencing the results obtained is the use of primary microplastics, i.e., those free of environmental contaminants. Environmental microplastics can be vectors for toxins, pathogens, and contaminants [28,41,42], and it is these that could potentially pose a greater threat than the microplastics themselves and cause adverse effects in humans and animals. However, more studies comparing the effects of environmental and primary microplastics are needed to prove this hypothesis. The use of microparticles exhibiting a wide variation in shape and size is aimed at approximating the natural conditions, as the actual exposure does not involve standardised particles. A dose of 0.1 g/animal/day is a dose that may indeed reflect daily life, whereas a dose of 1 g/animal/day actually exceeds real-life exposure to microplastics, according to available data [19,20]. However, its application is important in order to learn about the effects of a microplastic overdose.

There are reports available that indicate a high resistance of plastic particles to the action of artificial digestive juices [43], but Krasucka et al. demonstrated that under the influence of digestion, changes in the properties of plastics may occur, including the formation of nanoparticles [42]. Other studies also confirmed that MP-PET could reduce the number of bacterial groups responsible for maintaining the balance of the intestinal microflora and increase the number of bacterial groups capable of inducing a pro-inflammatory state [35]. Polyethylene (PE) microplastics were also observed to exhibit a synergistic action with *Helicobacter pylori*, thus contributing to gastric mucosal damage and inducing gastritis in mice [28]. Interestingly, the previously mentioned study suggests that the colonic microbiome could cause the biodegradation of polyethylene terephthalate [35]. It is known, however, that one of the most frequently described effects of exposure to nano- and microplastics is the induction of intestinal dysbiosis [10,13,40].

Peters et al. studied different types of microplastics (including PET) to find out which chemical substances and metals are released during chemical extraction and when using an in vitro human digestion model [44]. As for PET, the plasticisers bis(2-ethylhexyl) phthalate (DEHP), phthalic acid, di(2-propylpentyl) ester, and tributyl acetylcitrate were identified, as well as the metals lithium, manganese, cobalt, zirconium, tin, antimony, tantalum, and thallium [44]. The authors suggest that exposure to these substances through chemical wash-out when drinking bottled water is low [44], but other studies report that microplastics could be the main source of phthalates in the aqueous environment [45,46]. Further research is needed to determine the extent to which the release of plasticisers from microplastics poses a risk to plastic users.

Based on the available literature, it is known that microplastics can induce oxidative stress [10,13,27,29,39,40,47]. The induction of oxidative stress can be activated by different pathways, for example, by the TLR4/NOX2 signalling axis [48] or the NF-κB/NLRP3/IL-1β/MCLK pathway [49]. In a study on marine organisms, such as mussels, crustaceans, and fish, inhibition of acetylcholinesterase (AChE) activity, which is considered to be an indicator of neuromuscular toxicity [26], was observed. Exposure to PE in mice reduced the ACh levels in the brain tissue by, e.g., inducing oxidative stress [29]. ENS neurons are also sensitive to damage resulting from nitrosative stress, mitochondrial dysfunction, or the presence of inflammation [50]. Studies on mice assessing the effects of microplastics on the large intestinal mucosa demonstrated that they could contribute to the development of mild or moderate colitis, e.g., through intestinal dysbiosis, the occurrence of inflammatory infiltration in the mucosa, increased secretion of pro-inflammatory cytokines, the induction of apoptosis, a reduction in the number of goblet cells, mucin expression, the secretion of mucus, and increased intestinal barrier permeability or tissue accumulation, which corresponds with other available studies [10,13,26,40,41] and the results obtained in this study, especially in the HD group. Available data indicate that microplastics have the potential to exert multiple effects on the ENS as well as on the entire body [17,31,40,51]. The described mechanisms may be the cause of the changes observed in the study presented here.

Neurodegenerative and intestinal diseases pose a growing problem for society. Some of these diseases, e.g., Parkinson’s disease or Alzheimer’s disease, can have their origin in the ENS [52,53]. With age, under the influence of potentially toxic agents and changes in the intestinal microbiome, changes may occur in the population of ENS neurons and in the chemical coding of neurotransmitters, neuropeptides, and neuroimmunomodulators [52,53,54,55,56,57].

VIP, NO, and GAL exhibit neuroprotective action [58,59], but it should be noted that an excess of NO can form toxic compounds, namely reactive nitrogen species (RNS), which have already shown a neurodegenerative action [59,60]. Several mechanisms are involved in the neuroprotective properties of NO, including the activation of Akt and CREB (cAMP response element-binding protein) kinase, S-nitrosylation of the NR1 and NR2 subunits of the NMDA (N-methyl-D-aspartate) receptor, decreased Ca^2+^ ion influx, or increased haeme oxygenase expression [60,61].

NO has a similar effect on intestinal motility throughout the gastrointestinal tract, having a relaxing effect and reduces gastric acid secretion in the stomach, but in the duodenum and colon it stimulates the secretory function [59]. ACh stimulates the synthesis of NO, which is subsequently released from the submucosal arteriole endothelial cells, causing a relaxant effect on the vascular smooth muscles and thus regulating the blood flow [7]. Under the influence of bisphenol A [56], acetylsalicylic acid [55], acrylamide [54], and diabetes [62] in the jejunum, as in the present study, there was also a reduction in the percentage of nNOS-positive neurons, with the difference that all the three plexuses were affected. It has been noted that a reduction in the activity of nitric oxide synthase (NOS) occurs during hypoxic-ischaemic encephalopathy as a neuroprotective strategy [63]. Studies conducted on pigs to date also confirm that the highest concentration of nNOS-positive neurons is found in the myenteric plexus [55,56], which is also the case in this study. Interestingly, only in the case of nNOS-positive neurons, a dose of 0.1 g/day/animal (LD) was not observed to induce statistically significant changes in at least one of the plexuses, whereas a dose of 1 g/day/animal (HD) contributed to the induction of changes only in the MP and ISP, which may indicate, inter alia, an excessive loss of these neurons, e.g., through internal nitrosative damage [50], or a neuroprotective effect through the inhibition of nitric oxide activity. Research has also confirmed that one of the reasons for the reduction in immunoreactivity towards the test substance may be due to the degeneration or necrosis of fibres and neurons, which may be linked to the intensity of inflammation in the intestinal area [64].

The similarities noted in the populations of VIP- and nNOS-positive neurons in the MP may indicate the co-localisation of these neurons, especially as both of them serve a similar function in the regulation of intestinal motility [64,65], and, in addition, both of them may significantly affect neuronal survivability [66]. VIP exhibits neuroprotective action by maintaining a balance between pro- and anti-inflammatory cytokines [67], neuronal survivability [68,69], inhibiting the activity (phagocytosis and chemotaxis) of macrophages [70], stimulating the differentiation of B lymphocytes, and producing IgA [70,71], inhibiting the proliferation and migration of T lymphocytes [70,71], inhibiting the secretion of pro-inflammatory cytokines such as TNF-α, IFNγ, IL-6, IL-12 [69,70], increasing the secretion of IL-10 [69], and inhibiting the inducible nitric oxide synthase (iNOS) [70]. In the gastrointestinal tract, it causes the relaxation of smooth muscles and stimulates the secretion of mucus, intestinal juice, and bile [55,64,71,72]. The percentage of neurons often increases under the influence of inflammatory factors [55,56,67,69] and decreases, e.g., during Parkinson’s disease [73]. During inflammatory bowel disease (IBD), an increase or decrease in VIP was observed, depending on the study [53]. The lack of an increase in the percentage of nNOS- and VIP-positive neurons may reflect the fact that neither of these neurotransmitters is involved in the neuroprotection of neurons in the ENS under the influence of MP-PET.

Another substance for which a reduction in the percentage of positive neurons was noted is VAChT, a marker of cholinergic neurons. ACh is transported by VAChT to the synaptic vesicles and is present in the cholinergic nerve endings [7]. In the myenteric plexus, but only in the HD group, a reduced population of VAChT-positive neurons can be noted, which may result in the inhibition of muscle contractile activity, as ACh is considered to be the main substance inducing smooth muscle contractions [74]. Under the influence of both doses of MP-PET, there was a reduction in the percentage of VAChT-positive neurons in the OSP and ISP. This may result in reduced ion secretion [7,72], a lack of inhibition of pro-inflammatory cytokine secretion [68], or a lack of neuroprotective action [75]. During neurodegenerative diseases, both the loss of cholinergic neurons and a reduced percentage of VAChT neurons can be observed [76]. Inflammatory and toxic factors may, however, contribute to an increase [54] or a decrease in VAChT activity [56]. The neurotoxicity of nano- and microplastics has been described in animals such as invertebrates, fish, or rodents [26,29]. As for the studies mentioned, AChE activity was mainly inhibited [26]. A reduction in the percentage of VAChT-positive neurons may be another mechanism contributing to neurotoxicity.

The last substance for which a reduction in all the plexuses was noted is the neuropeptide CART. A study conducted on mice and pigs indicates that CART is primarily found in the stomach, the duodenum, and the jejunum [77]. Current data indicate that CART serves neuroprotective functions [55,77], has an effect on food intake, and reduces both the secretion of gastric juice and motor activity [54,74]. It was noted that in the jejunum, the population of CART-positive neurons was larger in the myenteric plexus than in the submucous plexuses, as reflected in the literature [55,56,78,79]. The population of CART-positive neurons in the jejunum increased under the influence of various agents, e.g., acrylamide [78], bisphenol A [56], and acetylsalicylic acid [55], or decreased in the Huntington’s disease [80]. In the duodenum, MP-PET also induced an increase in the population of CART-positive neurons [30]. A similar trend can be observed with the effect of bisphenol A on the small intestine, except that there was a decrease in the duodenum [79], whereas in the jejunum, there was an increase [56] in the percentage of CART-positive neurons.

The latter two substances exhibit increased immunoreactivity under the influence of MP-PET. GAL may exert anti-inflammatory action by increasing the synthesis of IFN-γ and IL-12/23 and decreasing TNF-α and IL-1β [56]. It regulates smooth muscle contraction [79], presumably by inhibiting ACh and SP [72], regulates the secretion and flow of the blood [81], and exhibits neurotrophic properties [67]. In myenteric neuron culture, GAL contributed to a decrease in neuronal survivability [68]. A study on diabetes observed that GAL contributed to the maintenance of glucose level homeostasis, could increase NO secretion, and inhibit ACh secretion [82]. The contractile activity is determined by the section of the gastrointestinal tract and the species under study [83]. In humans, GAL exhibited the same effectiveness in stimulating the contractile activity of the jejunum as ACh [83]. Increased GAL secretion can be observed when neuronal damage occurs [67,72]. It also exhibits a potential analgesic effect [84]. Under the influence of MP-PET, a shortening of the intestinal villi was observed in the HD group, which can potentially increase GAL expression as a response to tissue damage and the involvement in their regeneration [84]. If we consider GAL as a neuronal damage marker [72], the conclusion can be drawn that significant neuronal damage has occurred under the influence of MP-PET, and that this is the reason for the observed reduction in the populations of CART, VIP, VAChT, and nNOS neurons.

An increase in relation to both GAL and SP is often observed for agents likely to induce inflammation [54,56]. SP is a neuropeptide found in the intestinal neurons, which exhibits modulatory properties, e.g., through its action on the mast cells, macrophages, T and B lymphocytes, the plasmatic cells, and the nerve growth factor (NGF) [70,85]. In the submucous plexus, immune cells can be observed, including the mast cells [86], which can be stimulated to release histamine via, inter alia, SP, or calcitonin gene-related peptide (CGRP), which are among the major pro-inflammatory neuropeptides [2,85,87,88,89]. SP is involved in neurogenic inflammation, sensory conduction, and nociception [53,57,89,90]. SP, along with neuropeptide Y (NPY) and neurotensin, exhibits antimicrobial activity [3]. SP also pro-inflammatorily stimulates lymphocytes and macrophages, increases the production of the pro-inflammatory cytokines TNF-α, IL-1β, IL-6, and IL-12 [56,71,88], and is involved in inflammatory processes in the mucosa [53,70]. The inflammation they induce can cause changes in the secretion and intestinal motility patterns [64,87]. SP is often present in cholinergic neurons [65]. Both ACh and SP have a stimulatory effect on the intestinal muscle coat, as well as pro-secretory and vasodilatory effects [53,72,74,88,89,90]. SP exhibits a high affinity for the NK-1 receptor and a lower affinity for the NK-2 and NK-3 receptors [88]. It is the SP-NK-1 receptor that is involved in neuroimmune reactions in the intestines [88,89]. Through the same receptor, electrolyte and water secretion can also be stimulated in the small intestines and the colons of humans and animals [88,89,90]. The results obtained may reflect the fact that SP plays a lesser role in the regulation of small intestinal motility and a greater role in neuroprotective, immunological, and nociception processes. MP-PET can directly interact with the intestinal mucosa, which is why an increase in the percentage of SP-positive neurons was observed, especially in the ISP in LD and HD group. A similar response was noted in pigs exposed to *Schistosoma japonicum*, which indicates that more damage to the nerve plexus can be found closer to the intestinal lumen [64]. The aforementioned study observed that the VIP and SP levels varied depending on the severity of inflammation, but there was an increase noted in SP and a decrease in VIP in tissues with mild, moderate, and severe inflammation [64], which corresponds to the results obtained in the present study.

As the ENS is a complex system, a decrease in the percentage of CART-, nNOS-, VAChT-, and VIP-positive neurons and an increase in the percentage of SP- and GAL-positive neurons do not result in a zero-one response because there are other substances (e.g., serotonin, γ-aminobutyric acid (GABA), PACAP, NPY, CGRP, or dopamine), which were not the subject of the experiment described, that affect plasticity of the ENS [1,5,7,52,71,72]. It is noteworthy that no changes, such as a decrease in appetite, behavioural changes, or the occurrence of diarrhoea or growth inhibition (Figure 1), were clinically noted in the animals concerned throughout the duration of the experiment. It should be noted that the ENS is a complex biological system, and the results obtained represent only a fragment of knowledge on the effects of MP-PET on the domestic pig, and do not present other potential mechanisms that may be responsible for maintaining homeostasis of the entire system (and are likely to go beyond the ENS). It is, therefore, not possible to conclusively determine the ultimate nature of the impact of microplastics on the entire body.

The objective of this study was to identify statistically significant differences in neurotransmitter occurrence between the experimental groups, focusing on *p*-values derived from ANOVA with Scheffe’s post hoc test to indicate significant differences without addressing effect magnitudes. Due to the limited sample size, the reliability of effect size measures was potentially compromised, so the analysis prioritises *p*-values to highlight significant findings, with the understanding that future studies with larger samples will provide more robust effect size estimates. Given the complexity and exploratory nature of this investigation, the analysis was streamlined to focus on statistically significant differences, as calculating effect sizes for each neurotransmitter across multiple comparisons would complicate interpretation. Future studies should address these nuances in greater details.

## 5. Conclusions

The results obtained differ from the results obtained in previous studies [30]. This may be due to the different physicochemical properties of MP-PET and/or the neurotransmitters under study, depending on the section of the gastrointestinal tract. These differences may be due to the fact that MP-PET remains in the digestive tract longer, and is therefore under the influence of intestinal juice. The previously cited studies [42,44] show that digestion may cause changes in physicochemical properties, including the release of nanoparticles or chemical substances. These changes may potentially cause differences in the percentage of CART- and SP-positive neurons in the duodenum and jejunum under the influence of the same factor, MP-PET. Explaining the differences resulting from oral exposure to microplastics between the percentages of CART- and SP-positive neurons in different sections of digestive tract is problematic. The presented research results, according to the authors’ current knowledge, described studies are the first research focusing on microplastics, with this methodology and these animals. For this reason, it is difficult to discuss the potential cause of the differences between the percentage of CART and SP positive neurons in the duodenum and jejunum under the influence of MP-PET. Research results show that MP-PET affects miRNAs that target genes associated with lifestyle diseases (insulin resistance, obesity, cardiovascular disease, and cancer) [51]. Therefore, it may be assumed that it may also influence the expression of other genes or specific biochemical pathways [20]. Histological changes in both sections of the small intestine are comparable, but in the duodenum, a greater shortening of the villi was noted. It can therefore be assumed that in the duodenum, the physical aspect of MP-PET (particle size) may be more important, and in the jejunum, the chemical aspect and nanoparticles formed from MP-PET. The similarity in the structure of the gastrointestinal tract of domestic pigs and humans [6] proves that the present study provides valuable knowledge on the potential effects of oral exposure to microplastics on the human gastrointestinal tract, and guides further research into its effects on humans and animals.

Histological changes were more pronounced in the group receiving 1 g/animal/day of MP-PET. The same dose also contributed to the induction of an ENS response by increasing or decreasing the percentage of neurons positive for the tested substances. The only exception were nNOS neurons in OSP, where no changes occurred.

Based on this, it can be concluded that oral exposure to microplastics can affect the ENS function by neurotoxic and pro-inflammatory effects, yet there is a need for further research to determine the mechanism of this process and possible further effects. Based on the results obtained, the conclusion can be drawn to suggest that inflammation, mainly mediated by GAL and SP, is observed in the ENS of the porcine jejunum.

## Figures and Tables

**Table 1:** List of antibodies used in immunofluorescence method.

Antigen	Host Species	Final Dilution	Code	Supplier
**Primary Antibodies**				
Protein gene product 9.5	Mouse	1:1000	480012	ThermoFisher Scientific, Waltham, MA, USA
Neuronal nitric oxide synthase	Rabbit	1:3000	PA1-033	
Cocaine- and amphetamine-regulated transcript	Rabbit	1:16,000	H-003-61	Phoenix Pharmaceuticals, Inc., Burlingame, CA, USA
Vesicular acetylcholine transporter	Rabbit	1:2000	H-V006	
Galanin	Guinea pig	1:2000	T-5036	Peninsula, San Carlos, CA, USA
Substance P	Rat	1:150	8450-0505	BioRad, Hercules, CA, USA
Vasoactive intestinal peptide	Rabbit	1:2000	ab22736	Abcam, Cambridge, UK
**Secondary Antibodies**				
Alexa Fluor 488	Donkey anti-mouse IgG (H + L)	1:1000	A21202	ThermoFisher Scientific, Waltham, MA, USA
Alexa Fluor 546	Donkey anti-rabbit IgG (H + L)		A10040	
Alexa Fluor 546	Goat anti-guinea pig IgG (H + L)		A11074	
Alexa Fluor 546	Goat anti-rat IgG (H + L)		A11081	

## Data Availability

The data presented in this study are available on request from the corresponding author.

## References

[1] Fleming M.A., Ehsan L., Moore S.R., Levin D.E. (2020). The enteric nervous system and its emerging role as a therapeutic target. Gastroenterol. Res. Pract..

[2] Schneider S., Wright C.M., Heuckeroth R.O. (2019). Unexpected roles for the second brain: Enteric nervous system as master regulator of bowel function. Annu. Rev. Physiol..

[3] Green B.T., Brown D.R. (2010). Interactions between bacteria and the gut mucosa: Do enteric neurotransmitters acting on the mucosal epithelium influence intestinal colonization or infection?. Microbial Endocrinology: Interkingdom Signaling in Infectious Disease and Health.

[4] Shahrestani J., Das J.M. (2023). Neuroanatomy, auerbach plexus. StatPearls [Internet].

[5] Furness J.B. (2000). Types of neurons in the enteric nervous system. J. Auton. Nerv. Syst..

[6] Mazzoni M., Caremoli F., Cabanillas L., de Los Santos J., Million M., Larauche M., Clavenzani P., De Giorgio R., Sternini C. (2021). Quantitative analysis of enteric neurons containing choline acetyltransferase and nitric oxide synthase immunoreactivities in the submucosal and myenteric plexuses of the porcine colon. Cell Tissue Res..

[7] Harrington A.M., Hutson J.M., Southwell B.R. (2010). Cholinergic neurotransmission and muscarinic receptors in the enteric nervous system. Prog. Histochem. Cyto..

[8] Schemann M., Neunlist M. (2004). The human enteric nervous system. Neurogastroenterol. Motil..

[9] Karaki S.I., Kuwahara A. (2004). Regulation of intestinal secretion involved in the interaction between neurotransmitters and prostaglandin E2. Neurogastroenterol. Motil..

[10] Huang Z., Weng Y., Shen Q., Zhao Y., Jin Y. (2021). Microplastic: A potential threat to human and animal health by interfering with the intestinal barrier function and changing the intestinal microenvironment. Sci. Total Environ..

[11] Prata J.C., da Costa J.P., Lopes I., Andrady A.L., Duarte A.C., Rocha-Santos T. (2021). A One Health perspective of the impacts of microplastics on animal, human and environmental health. Sci. Total Environ..

[12] Dong X., Liu X., Hou Q., Wang Z. (2023). From natural environment to animal tissues: A review of microplastics (nanoplastics) translocation and hazards studies. Sci. Total Environ..

[13] Ali N., Katsouli J., Marczylo E.L., Gant T.W., Wright S., de la Serna J.B. (2024). The potential impacts of micro-and-nano plastics on various organ systems in humans. EBioMedicine.

[14] Pinlova B., Nowack B. (2024). From cracks to secondary microplastics-surface characterization of polyethylene terephthalate (PET) during weathering. Chemosphere.

[15] Al Mamun A., Prasetya T.A.E., Dewi I.R., Ahmad M. (2023). Microplastics in human food chains: Food becoming a threat to health safety. Sci. Total Environ..

[16] Matias R.S., Gomes S., Barboza L.G.A., Salazar-Gutierrez D., Guilhermino L., Valente L.M. (2023). Microplastics in water, feed and tissues of European seabass reared in a recirculation aquaculture system (RAS). Chemosphere.

[17] Zhu L., Kang Y., Ma M., Wu Z., Zhang L., Hu R., Xu Q., Zhu J., Gu X., An L. (2024). Tissue accumulation of microplastics and potential health risks in human. Sci. Total Environ..

[18] Pironti C., Ricciardi M., Motta O., Miele Y., Proto A., Montano L. (2021). Microplastics in the environment: Intake through the food web, human exposure and toxicological effects. Toxics.

[19] Senathirajah K., Attwood S., Bhagwat G., Carbery M., Wilson S., Palanisami T. (2021). Estimation of the mass of microplastics ingested–A pivotal first step towards human health risk assessment. J. Hazard. Mater..

[20] Grodzicki W., Dziendzikowska K., Gromadzka-Ostrowska J., Kruszewski M. (2021). Nanoplastic impact on the gut-brain axis: Current knowledge and future directions. Int. J. Mol. Sci..

[21] Yang Z., Wang M., Feng Z., Wang Z., Lv M., Chang J., Chen L., Wang C. (2023). Human Microplastics Exposure and Potential Health Risks to Target Organs by Different Routes: A Review. Curr. Pollut. Rep..

[22] Xu J., Bi W., Hua L., Cheng Z., Wang Y., Li D., Liu W., Wang L., Sun H. (2022). Wide occurrence of seven phthalate plasticizers and two typical microplastics in pig feed. Chemosphere.

[23] Yang J., Li R., Zhou Q., Li L., Li Y., Tu C., Zhao X., Xiong K., Christie P., Luo Y. (2021). Abundance and morphology of microplastics in an agricultural soil following long-term repeated application of pig manure. Environ. Pollut..

[24] Habib R.Z., Poulose V., Alsaidi R., Al Kendi R., Iftikhar S.H., Mourad A.-H.I., Kittaneh W.F., Thiemann T. (2022). Plastic cutting boards as a source of microplastics in meat. Food Addit. Contam. Part A.

[25] Sánchez A., Rodríguez-Viso P., Domene A., Orozco H., Vélez D., Devesa V. (2022). Dietary microplastics: Occurrence, exposure and health implications. Environ. Res..

[26] Prüst M., Meijer J., Westerink R.H. (2020). The plastic brain: Neurotoxicity of micro-and nanoplastics. Part. Fibre Toxicol..

[27] Ding R., Chen Y., Shi X., Li Y., Yu Y., Sun Z., Duan J. (2024). Size-dependent toxicity of polystyrene microplastics on the gastrointestinal tract: Oxidative stress related-DNA damage and potential carcinogenicity. Sci. Total Environ..

[28] Tong X., Li B., Li J., Li L., Zhang R., Du Y., Zhang Y. (2022). Polyethylene microplastics cooperate with Helicobacter pylori to promote gastric injury and inflammation in mice. Chemosphere.

[29] Wang S., Han Q., Wei Z., Wang Y., Xie J., Chen M. (2022). Polystyrene microplastics affect learning and memory in mice by inducing oxidative stress and decreasing the level of acetylcholine. Food Chem. Toxicol..

[30] Gałęcka I., Szyryńska N., Całka J. (2024). Influence of polyethylene terephthalate (PET) microplastic on selected active substances in the intramural neurons of the porcine duodenum. Part. Fibre Toxicol..

[31] Deng Y., Zhang Y., Lemos B., Ren H. (2017). Tissue accumulation of microplastics in mice and biomarker responses suggest widespread health risks of exposure. Sci. Rep..

[32] Ma Y., Xu D., Wan Z., Wei Z., Chen Z., Wang Y., Han X., Chen Y. (2024). Exposure to different surface-modified polystyrene nanoparticles caused anxiety, depression, and social deficit in mice via damaging mitochondria in neurons. Sci. Total Environ..

[33] Xiong F., Liu J., Xu K., Huang J., Wang D., Li F., Wang S., Zhang J., Pu Y., Sun R. (2023). Microplastics induce neurotoxicity in aquatic animals at environmentally realistic concentrations: A meta-analysis. Environ. Pollut..

[34] Qiao R., Deng Y., Zhang S., Wolosker M.B., Zhu Q., Ren H., Zhang Y. (2019). Accumulation of different shapes of microplastics initiates intestinal injury and gut microbiota dysbiosis in the gut of zebrafish. Chemosphere.

[35] Tamargo A., Molinero N., Reinosa J.J., Alcolea-Rodriguez V., Portela R., Bañares M.A., Fernández J.F., Moreno-Arribas M.V. (2022). PET microplastics affect human gut microbiota communities during simulated gastrointestinal digestion, first evidence of plausible polymer biodegradation during human digestion. Sci. Rep..

[36] Xu M., Halimu G., Zhang Q., Song Y., Fu X., Li Y., Li Y., Zhang H. (2019). Internalization and toxicity: A preliminary study of effects of nanoplastic particles on human lung epithelial cell. Sci. Total Environ..

[37] Stock V., Laurisch C., Franke J., Dönmez M.H., Voss L., Böhmert L., Braeuning A., Sieg H. (2021). Uptake and cellular effects of PE, PP, PET and PVC microplastic particles. Toxicol. In Vitro.

[38] Bruinink A., Wang J., Wick P. (2015). Effect of particle agglomeration in nanotoxicology. Arch. Toxicol..

[39] Stock V., Böhmert L., Lisicki E., Block R., Cara-Carmona J., Pack L.K., Selb R., Lichtenstein D., Voss L., Henderson C.J. (2019). Uptake and effects of orally ingested polystyrene microplastic particles in vitro and in vivo. Arch. Toxicol..

[40] Hirt N., Body-Malapel M. (2020). Immunotoxicity and intestinal effects of nano-and microplastics: A review of the literature. Part. Fibre Toxicol..

[41] Zolotova N., Dzhalilova D., Tsvetkov I., Makarova O. (2023). Influence of Microplastics on Morphological Manifestations of Experimental Acute Colitis. Toxics.

[42] Krasucka P., Bogusz A., Baranowska-Wójcik E., Czech B., Szwajgier D., Rek M., Ok Y.S., Oleszczuk P. (2022). Digestion of plastics using in vitro human gastrointestinal tract and their potential to adsorb emerging organic pollutants. Sci. Total Environ..

[43] Stock V., Fahrenson C., Thuenemann A., Dönmez M.H., Voss L., Böhmert L., Braeuning A., Lampen A., Sieg H. (2020). Impact of artificial digestion on the sizes and shapes of microplastic particles. Food Chem. Toxicol..

[44] Peters R., de Jong N., de Haan L., Wright S., Bouwmeester H. (2022). Release and intestinal translocation of chemicals associated with microplastics in an in vitro human gastrointestinal digestion model. Microplast. Nanoplast..

[45] Cao Y., Lin H., Zhang K., Xu S., Yan M., Leung K.M., Lam P.K. (2022). Microplastics: A major source of phthalate esters in aquatic environments. J. Hazard. Mater..

[46] Zhong X., Yi X., Cheng F., Tong H., Xu W., Yang X. (2023). Leaching of di-2-ethylhexyl phthalate from biodegradable and conventional microplastics and the potential risks. Chemosphere.

[47] Li Z., Chang X., Hu M., Fang J.K.-H., Sokolova I.M., Huang W., Xu E.G., Wang Y. (2022). Is microplastic an oxidative stressor? Evidence from a meta-analysis on bivalves. J. Hazard. Mater..

[48] Wu H., Xu T., Chen T., Liu J., Xu S. (2022). Oxidative stress mediated by the TLR4/NOX2 signalling axis is involved in polystyrene microplastic-induced uterine fibrosis in mice. Sci. Total Environ..

[49] Zeng G., Li J., Wang Y., Su J., Lu Z., Zhang F., Ding W. (2024). Polystyrene microplastic-induced oxidative stress triggers intestinal barrier dysfunction via the NF-κB/NLRP3/IL-1β/MCLK pathway. Environ. Pollut..

[50] Stavely R., Ott L.C., Rashidi N., Sakkal S., Nurgali K. (2023). The Oxidative Stress and Nervous Distress Connection in Gastrointestinal Disorders. Biomolecules.

[51] Mierzejewski K., Kurzyńska A., Golubska M., Całka J., Gałęcka I., Szabelski M., Paukszto Ł., Andronowska A., Bogacka I. (2023). New insights into the potential effects of PET microplastics on organisms via extracellular vesicle-mediated communication. Sci. Total Environ..

[52] Nguyen T.T., Baumann P., Tüscher O., Schick S., Endres K. (2023). The Aging Enteric Nervous System. Int. J. Mol. Sci.

[53] Vasina V., Barbara G., Talamonti L., Stanghellini V., Corinaldesi R., Tonini M., De Ponti F., De Giorgio R. (2006). Enteric neuroplasticity evoked by inflammation. Auton. Neurosci..

[54] Bulc M., Całka J., Palus K. (2022). Administration of Different Doses of Acrylamide Changed the Chemical Coding of Enteric Neurons in the Jejunum in Gilts. Int. J. Environ. Res. Public Health.

[55] Rząp D., Czajkowska M., Całka J. (2020). Neurochemical plasticity of nNOS-, VIP-and CART-immunoreactive neurons following prolonged acetylsalicylic acid supplementation in the porcine jejunum. Int. J. Mol. Sci.

[56] Szymanska K., Gonkowski S. (2019). Neurochemical characterization of the enteric neurons within the porcine jejunum in physiological conditions and under the influence of bisphenol A (BPA). Neurogastroenterol. Motil..

[57] Gonkowski S., Gajęcka M., Makowska K. (2020). Mycotoxins and the enteric nervous system. Toxins.

[58] Bulc M., Całka J., Palus K. (2023). Changes in the Phenotype of Intramural Inhibitory Neurons of the Porcine Descending Colon Resulting from Glyphosate Administration. Int. J. Mol. Sci..

[59] Szymanska K., Calka J., Gonkowski S. (2018). Nitric oxide as an active substance in the enteric neurons of the porcine digestive tract in physiological conditions and under intoxication with bisphenol A (BPA). Nitric Oxide.

[60] Calabrese V., Mancuso C., Calvani M., Rizzarelli E., Butterfield D.A., Giuffrida Stella A.M. (2007). Nitric oxide in the central nervous system: Neuroprotection versus neurotoxicity. Nat. Rev. Neurosci..

[61] Tse J.K. (2017). Gut microbiota, nitric oxide, and microglia as prerequisites for neurodegenerative disorders. ACS Chem. Neurosci..

[62] Bulc M., Palus K., Dąbrowski M., Całka J. (2019). Hyperglycaemia-induced downregulation in expression of nNOS intramural neurons of the small intestine in the pig. Int. J. Mol. Sci..

[63] Favié L., Cox A.R., Groenendaal F. (2018). Nitric oxide synthase inhibition as a neuroprotective strategy following hypoxic–ischemic encephalopathy: Evidence from animal studies. Front. Neurol..

[64] Balemba O., Semuguruka W., Hay-Schmidt A., Johansen M., Dantzer V. (2001). Vasoactive intestinal peptide and substance P-like immunoreactivities in the enteric nervous system of the pig correlate with the severity of pathological changes induced by Schistosoma japonicum. Int. J. Parasitol..

[65] Makowska K., Gonkowski S. (2023). Changes Caused by Bisphenols in the Chemical Coding of Neurons of the Enteric Nervous System of Mouse Stomach. Int. J. Environ. Res. Public Health.

[66] Sandgren K., Lin Z., Svenningsen Å.F., Ekblad E. (2003). Vasoactive intestinal peptide and nitric oxide promote survival of adult rat myenteric neurons in culture. J. Neurosci. Res..

[67] Zacharko-Siembida A., Piedra J.L.V., Szymańczyk S., Arciszewski M.B. (2013). Immunolocalization of NOS, VIP, galanin and SP in the small intestine of suckling pigs treated with red kidney bean (*Phaseolus vulgaris*) lectin. Acta Histochem..

[68] Arciszewski M.B., Ekblad E. (2005). Effects of vasoactive intestinal peptide and galanin on survival of cultured porcine myenteric neurons. Regul. Pept..

[69] Ekblad E., Bauer A. (2004). Role of vasoactive intestinal peptide and inflammatory mediators in enteric neuronal plasticity. Neurogastroenterol. Motil..

[70] Janjatović A.K., Valpotić H., Kezić D., Lacković G., Gregorović G., Sladoljev S., Mršić G., Popović M., Valpotić I. (2012). Secretion of immunomodulating neuropeptides (VIP, SP) and nitric oxide synthase in porcine small intestine during postnatal development. Eur. J. Histochem..

[71] Genton L., Kudsk K.A. (2003). Interactions between the enteric nervous system and the immune system: Role of neuropeptides and nutrition. Am. J. Surg..

[72] Nezami B.G., Srinivasan S. (2010). Enteric nervous system in the small intestine: Pathophysiology and clinical implications. Curr. Gastroenterol. Rep..

[73] Giancola F., Torresan F., Repossi R., Bianco F., Latorre R., Ioannou A., Guarino M., Volta U., Clavenzani P., Mazzoni M. (2017). Downregulation of neuronal vasoactive intestinal polypeptide in Parkinson’s disease and chronic constipation. Neurogastroenterol. Motil..

[74] Burliński P.J., Rychlik A., Całka J. (2014). Effects of inflammation and axotomy on expression of acetylcholine transferase and nitric oxide synthetase within the cocaine-and amphetamine-regulated transcript-immunoreactive neurons of the porcine descending colon. J. Comp. Pathol..

[75] Szymanska K., Makowska K., Gonkowski S. (2018). The influence of high and low doses of bisphenol A (BPA) on the enteric nervous system of the porcine ileum. Int. J. Mol. Sci..

[76] Pepeu G., Giovannini M.G. (2017). The fate of the brain cholinergic neurons in neurodegenerative diseases. Brain Res..

[77] Ekblad E. (2006). CART in the enteric nervous system. Peptides.

[78] Palus K., Makowska K., Całka J. (2018). Acrylamide-induced alterations in the cocaine-and amphetamine-regulated peptide transcript (CART)-like immunoreactivity within the enteric nervous system of the porcine small intestines. Ann. Anat..

[79] Szymanska K., Gonkowski S. (2018). Bisphenol A—Induced changes in the enteric nervous system of the porcine duodenum. Neurotoxicology.

[80] van der Burg J.M., Winqvist A., Aziz N.A., Maat-Schieman M.L., Roos R.A., Bates G.P., Brundin P., Björkqvist M., Wierup N. (2011). Gastrointestinal dysfunction contributes to weight loss in Huntington’s disease mice. Neurobiol. Dis..

[81] Di Giancamillo A., Vitari F., Bosi G., Savoini G., Domeneghini C. (2010). The chemical code of porcine enteric neurons and the number of enteric glial cells are altered by dietary probiotics. Neurogastroenterol. Motil..

[82] Abot A., Lucas A., Bautzova T., Bessac A., Fournel A., Le-Gonidec S., Valet P., Moro C., Cani P.D., Knauf C. (2018). Galanin enhances systemic glucose metabolism through enteric Nitric Oxide Synthase-expressed neurons. Mol. Metab..

[83] Bálint A., Fehér E., Kisfalvi I., Máté M., Zelles T., Vizi E.S., Varga G. (2001). Functional and immunocytochemical evidence that galanin is a physiological regulator of human jejunal motility. J. Physiol. Paris.

[84] Wiesenfeld-Hallin Z., Xu X.-J. (2001). Neuropeptides in neuropathic and inflammatory pain with special emphasis on cholecystokinin and galanin. Eur. J. Pharmacol..

[85] Pidsudko Z., Kaleczyc J., Wąsowicz K., Sienkiewicz W., Majewski M., Zając W., Łakomy M. (2008). Distribution and chemical coding of intramural neurons in the porcine ileum during proliferative enteropathy. J. Comp. Pathol..

[86] Ostertag D., Buhner S., Michel K., Pehl C., Kurjak M., Götzberger M., Schulte-Frohlinde E., Frieling T., Enck P., Phillip J. (2015). Reduced responses of submucous neurons from irritable bowel syndrome patients to a cocktail containing histamine, serotonin, TNFα, and tryptase (IBS-cocktail). Front. Neurosci..

[87] Xue J., Askwith C., Javed N.H., Cooke H.J. (2007). Autonomic nervous system and secretion across the intestinal mucosal surface. Auton. Neurosci..

[88] Koon H.W., Pothoulakis C. (2006). Immunomodulatory properties of substance P: The gastrointestinal system as a model. Ann. N. Y. Acad. Sci..

[89] Harrison S., Geppetti P. (2001). Substance p. Int. J. Biochem. Cell Biol..

[90] Gonkowski S. (2013). Substance P as a neuronal factor in the enteric nervous system of the porcine descending colon in physiological conditions and during selected pathogenic processes. Biofactors.

